# Internet of Robotic Things: Current Technologies, Challenges, Applications, and Future Research Topics

**DOI:** 10.3390/s25030765

**Published:** 2025-01-27

**Authors:** Jakub Krejčí, Marek Babiuch, Jiří Suder, Václav Krys, Zdenko Bobovský

**Affiliations:** 1 Department of Robotics, VSB—Technical University of Ostrava, 708 00 Ostrava, Czech Republic; vaclav.krys@vsb.cz (V.K.); zdenko.bobovsky@vsb.cz (Z.B.); 2 Department of Control Systems and Instrumentation, VSB—Technical University of Ostrava, 708 00 Ostrava, Czech Republic; 3 Department of Computer Science, Electrical Engineering and Mathematical Sciences, Western Norway University of Applied Sciences, 6812 Førde, Norway; jiri.suder@hvl.no

**Keywords:** IoRT, industry 4.0, industry 5.0, robot, robotic things, industrial applications, cloud-based, smart factories

## Abstract

This article focuses on the integration of the Internet of Things (IoT) and the Internet of Robotic Things, representing a dynamic research area with significant potential for industrial applications. The Internet of Robotic Things (IoRT) integrates IoT technologies into robotic systems, enhancing their efficiency and autonomy. The article provides an overview of the technologies used in IoRT, including hardware components, communication technologies, and cloud services. It also explores IoRT applications in industries such as healthcare, agriculture, and more. The article discusses challenges and future research directions, including data security, energy efficiency, and ethical issues. The goal is to raise awareness of the importance of IoRT and demonstrate how this technology can bring significant benefits across various sectors.

## 1. Introduction

The Internet of Robotic Things (IoRT) is one of several branches of the Internet of Things (IoT), utilizing advancements in smart sensors, communication technologies, and internet protocols [1], as well as technologies like wireless sensor networks and cloud computing [2]. This is similar to the Industrial Internet of Things [3], the Internet of Medical Things for monitoring vital signs [4], smart e-Health gateways [5], telemedicine platforms [6], the Internet of Agricultural Things for precision farming [7], food supply chain management [8], environmental monitoring [9], or the Internet of Military Things for battlefield awareness [10]. All of them are connected in some sense, from the very edge—where they collect the most accurate data directly at the source, the so-called “Edge” [11]—through the Internet fog [12], which is made up of millions of paths consisting of different communication protocols, referred to in [13], to a central system where computation is performed, mostly a cloud platform [14]. All these sectors together form a global system called the Internet of Everything [15]. The name IoRT already implies that it is an application of IoT on robotic systems. The field of robotics is itself a very dynamic field in terms of development and implementation. IoT in this field, therefore, serves to make the individual systems even more efficient, and thus completes the modern notion of autonomous robots [16] and intelligent manufacturing systems [17] that complete the picture of smart factories and industry 4.0 [18]. It is widely acknowledged that the benefits of IoT in individual industries are large, and these systems are expected to spread to other areas as well, though the basis is very similar for all areas.

Our article will therefore present characteristics typical of the IoRT area, with a focus on industrial and mobile robotics. This is due to the constant evolution of the devices, systems, and principles used. There is also a need to raise awareness of this issue so that it is better understood and thus easier to introduce into the field of robotics. Furthermore, it is essential to emphasize the significance of the topic and demonstrate that the introduction of the IoRT offers substantially more benefits than drawbacks.

The article describes the technologies used in IoRT, focusing on the use of hardware as one of the main elements in IoT. This includes sensors, actuators, microcomputers, and microcontrollers. These components are used as the basis for small IoT devices, such as those for air quality monitoring [19]. Additionally, it covers other elements that enable communication and data collection between the robotic system and the IoRT infrastructure. It also discusses communication technologies as a key element that enables data transfer between the robotic system and the cloud platform. In addition, it includes the inclusion of these technologies in a layered scheme, for which this article [20] describes the basics. Cloud services and their inter-comparison are also described, as well as additional software used for data management and evaluation. Among other things, the content of this study is a description of AI technologies and their use in industrial robotics, with a link to IoT technologies, which, from our point of view, is crucial for further streamlining production processes.

Part of the article is also devoted to a description of the architectures of the technologies described in it. It is a description of the principle of operation of sensors and actuators and a description of how they are implemented in robotic systems. Another important area is the architecture of communication protocols, which describes how data are transferred. Why and how data are encrypted is described in this article [21]. This is followed by the area related to security architecture, which is a big topic in IoT and its subordinate domains and is further explored in the paper.

The theoretical part then goes into a part where specific applications of these systems in robotics are described. After the theoretical background, this section will give the reader a concrete idea of the applications in the field of robotics. In this section, the paper focuses on the use of IoT in collaborative robotics, whose use in Industry 4.0 is discussed in this article [22], where the systems are mainly used to monitor the space and to secure the work between human and robot. The next section then focuses on the concepts of “Big data” and “Big data management”, which are both described in this article [23], and the long-term monitoring of the collected data for their evaluation and so-called “predictive maintenance”, an interesting area thanks to which it is possible to predict and prevent possible situations. A section is also devoted to smart factories and flexible factories, as extensively covered in this review article [24], and their impact on competitiveness and environmental friendliness in industry. IoRT also has a considerable impact on the functioning of autonomous robots, for which a comparison can be found, for example, in the area dedicated to autonomous vehicles [25], which, from a certain perspective, are also robotic systems.

In the final part of the article, we touch upon the most important topic from our point of view, and that is what IoRT can bring in the future, and what will still need to be dealt with. The main points are described and explained so that the reader can get an idea of both the complexity of the issue and also what it will offer and how it will move the field of robotics forward.

While the importance of the described topic is evident, it is still a young area in which many things are still unexplored, and yet it already has huge potential and many things to offer. In many ways, by observing the benefits of IoT in other fields, such as healthcare, agriculture, and transportation [26], we can find inspiration for applications directly in robotic systems. At the same time, by comparing it to these other fields, we can imagine how significant the impact of IoT will be in robotics. The trend is set and we just need to understand and apply it correctly to make robotic systems more efficient, which ultimately makes our lives better.

## 2. Relevance of the Topic

**Figure 1:**
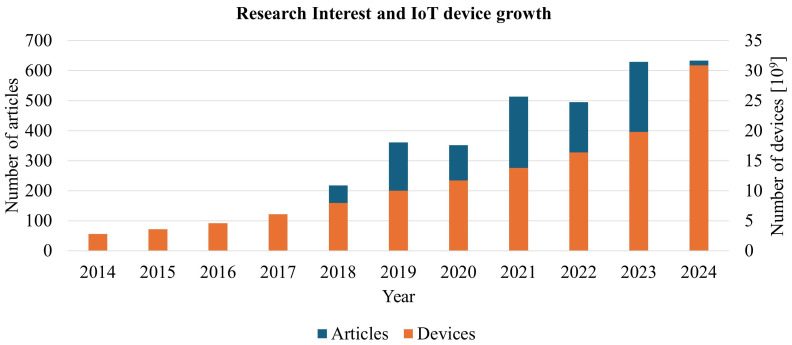
Research interest based on the number of publications in each year.

The topic of robotics and the Internet of Things (IoT) has gained significant traction in the academic community, as evidenced by a steady increase in scholarly publications over recent years. This upward trend reflects the growing interest in and importance of these technologies, both in theoretical research and practical applications. Researchers are exploring diverse areas such as machine learning integration, autonomous systems, and IoT-enabled robotics, leading to a surge in papers that address innovative methodologies, case studies, and emerging challenges. The consistent rise in academic output underscores the relevance and urgency of these fields, indicating that robotics and IoT are not just pivotal research areas but also key drivers of future technological progress and interdisciplinary collaboration. In the Figure 1, we present data demonstrating the stable growth of both IoT device deployments and scientific interest in this domain. This figure is based on data from Scopus, reflecting academic publications, and from IoT Analytics, providing insights into the growth of connected devices. To obtain the data, two keywords—IoT and Robot—were used in the Scopus search to include as many articles as possible that combine these two areas. Articles with no significant narrative value or articles that do not address the issue were then excluded from the list, and a total of 3409 articles from 2014 were included in the survey. The combined data sources clearly illustrate the parallel expansion of the IoT landscape in both practical implementation and scholarly investigation. The survey, based on the number of publications registered each year in Scopus, further reinforces this observation.

## 3. Fundamentals of IoRT

**Figure 2:**
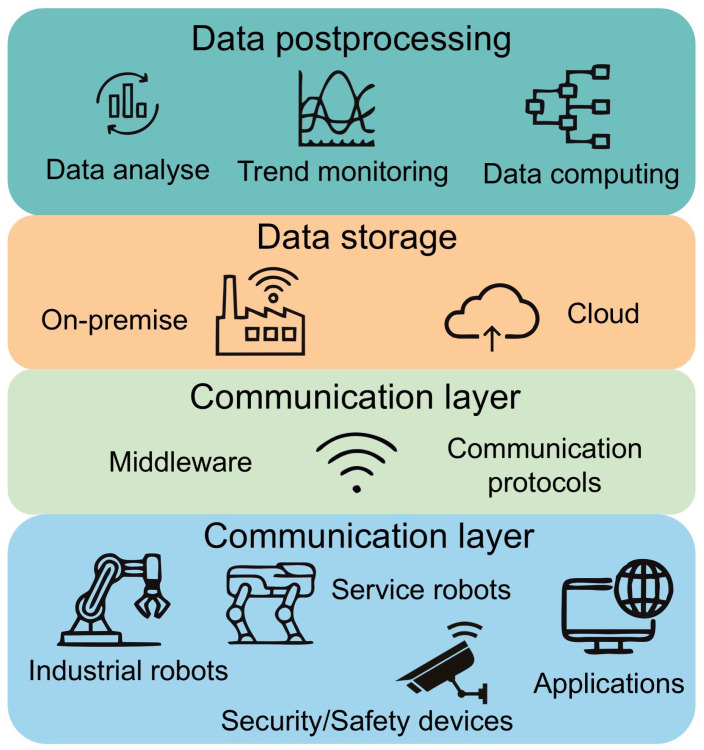
Placing robotic things at the lowest level of the layered model of IoRT logic.

Given the breadth of the topic, there are also a large number of different technologies applicable in the IoRT sphere [27]. These technologies can be simply divided into a group containing hardware tools and a group containing software tools [28]. Besides the obvious hardware components, such as sensors and actuators, we can also include data and information processing tools in the hardware group [29]. These are mainly computers, microcomputers, and microcontrollers. Furthermore, we also include robotic “things” as such in this group, which, for us, are the lowest layer in the layered logic (see Figure 2). These represent the subject under investigation. In this section, we discuss these robotic entities in detail and describe their placement in the hierarchy of robotic systems and their relation to the Internet of Things. An important part is then the systems for data storage and specifically for the storage and processing of large amounts of data. These will also be touched upon in the section. The second group, the group of software tools, includes everything else. These are all tools that enable communication in the wireless IoT environment: tools that take care of the security of the data being sent; tools that take care of the reliable delivery of data to the target area; and tools and systems that take care of data mining. We will describe these tools using the concept of a layered model. Another big area is all the software tools that we use to process the collected data. Last but not least, AI tools and their interconnection to industry, industrial robotics, and the concept of Industry 4.0 must be included in this group. The section also discusses selected programming languages that are used in IoRT. This section is important to understand the processes that take place throughout the network. As a result, the reader will be better able to identify and select technologies for specific applications on robotic systems.

### 3.1. Robotic Things

**Figure 3:**
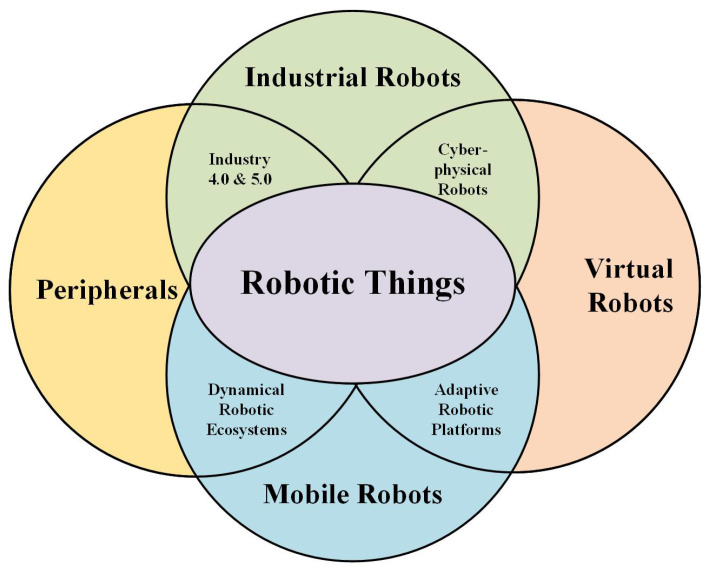
Distribution of the Robotic Things.

Generally, the term “Thing” in IoT refers to a physical object that generates data [30]. This can be, for example, weather stations, security systems, or even a mobile phone. In the context of IoRT, “Robotic Things” is a physical device that consists of small “Things”, which may be called subsystems [29]. These can then be put together to form complex mechatronic systems such as industrial robots or, for example, drones and other service robots [31]. These are also systems that can react to changes in the environment and communicate with other systems. Robotic systems stand out for their large range of deployment, while also offering a large number of tasks that they can perform. These tasks can be complex and complicated, such as driving an autonomous vehicle [32], or they can be simpler repetitive pick and place operations [33]. Depending on the deployment of the robot, the mechatronic system will then come into contact with different environments. Robotic systems today are used by industry, healthcare, transportation, agriculture, smart homes, security, and many others [34]. The number of subsystems is then linked to the complexity of the task and the environment in which the robotic system is deployed. Due to the complexity, we decided to categorize the robotic systems into four groups—industrial robots, mobile robots, virtual robots, and peripherals—as shown in Figure 3, which describes the interconnection of these IoRT areas and how they relate to each other.

#### 3.1.1. Industrial Robots

The basic division of robotic systems is usually into service robots or mobile robots and industrial robots. We understand an industrial robot as a complex mechatronic system designed to perform tasks intensively and automatically. The characteristics of an industrial robot depend largely on the application for which they are applied, the environment in which they will operate and finally the construction of the robot itself. However, industrial robots with universal use are usually used from an economic perspective [35]. The robot placement environment will be mostly static, where the robot is fixed in a workcell and where only the manipulation object changes. An industrial robot then can also be placed in a dynamically changing environment, where its location can be on a moving platform and the robot interacts with a multitude of external influences. In this environment, a collision with a human may also occur. The article [36], for example, describes these areas within disassembly processes where robots and humans work together. Another article [37] focuses on the safety risks in human–robot collaboration and proposes a safety strategy to reduce these risks. A general overview is provided by the article [38], which focuses on the current knowledge in this area within smart manufacturing processes. Dynamically changing environments are then more interesting from the point of view of data collection and applicability of IoT systems, which can bring many improvements here. For instance, in this paper [39], the authors look at the effect of robot parameters such as speed and acceleration temperature on the energy consumed by the robot. An industrial robot consists of individual subsystems and, as mentioned in the previous section, these subsystems can be viewed as individual things in the IoT concept. The first subsystem is the mechanical structure of the robot described, for example, in this article [40]. Today, we know a large number of mechanical structures of industrial robots, where the differences are mainly found in the number of degrees of freedom (DOF) and the working space of the robot. The most widely used type of industrial robot today is the articulated robot, due to its universal design with 6 DOF [41]. The mechanical structure affects the robot’s accuracy in performing tasks. The trend in the development of these robotic arms today is towards less robust designs and the use of non-traditional materials [42]. This can be, for example, composite materials used in combination with 3D printed parts. The authors of this article [43] offer a comprehensive insight for assembling a robotic arm from 3D printed parts. The mechanical structure of industrial robot is closely related to the stiffness of the robot and the vibration of the whole system [44]. For effective robot control and management, it is essential to have an accurate dynamic model. Depending on the type of task, the accuracy is more or less negligible. For tasks such as pick and place operations [45], there is no need to consider high accuracy; on the other hand, for tasks such as machining, we need the most accurate robot performance. A closer look at this is given in this paper [46], in which different approaches to increase the accuracy of an industrial robot in machining are selected. Another subsystem is the control and monitoring subsystem. These subsystems involve real-time data collection, diagnosis, and parameter visualization. They are essential for monitoring the robot’s performance and ensuring smooth operation; you can read more about them in this article [47].

#### 3.1.2. Mobile Robots

Mobile robotic systems are basically systems that are not permanently connected to a power source. They use batteries and supplementary—not main—energy sources, such as solar panels, to function. However, batteries are still their main source of energy, even though the drive for independence from them is on the rise. Advantageous to this effort are, for example, autonomous aircraft operating at high altitudes, where the effort is both research-based [48] and commercial. For example, [49] from Airbus is developing this high-altitude platform station with an operating time of up to several months. Having defined mobile robots in relation to others, they can then be further divided. We can divide them according to the way they move, according to the way they navigate, according to the environment, and finally, we can divide them according to their use and deployment [50]. These areas are closely related and it is advisable to define them precisely before designing a mobile robot. In general, we divide robots according to their movement into wheeled robots, i.e., robots that use a wheeled chassis to move in the environment [51]. An interesting group of robots with wheeled chassis are robots using omniwheel. This paper [52] describes the design of such a type of robot equipped with an omniwheel, and another article [53] describes their use. We then have crawler robots, walking robots, floating robots, and flying robots—the aforementioned drones. According to the method of navigation, we then distinguish between autonomous robots or remotely controlled robots, or a combination of both, where the operator has control and the robot—and the robot only—helps the operator in certain situations. Based on the environment, we can divide robots into ground, underwater, and aerial robots, but also into robots moving indoors or outdoors. Robots operating in confined spaces can also be distinguished by the chemical composition of the air around the robot. Safe motion and control of the robot in different environments is the focus of this study [54].

#### 3.1.3. Virtual Robots in Industry

Virtual robotic systems are software agents [55] that operate in a digital environment and that perform tasks such as processing information, communicating with the user, or automating certain processes. Today, they can be found in almost every application we use, even in everyday life [56]. Unlike the robotic systems mentioned earlier, these robotic systems do not have a physical form and their functioning is at the level of the software environment. We divide them based on the level of autonomy, function, and the environment for which they are run. According to autonomy, we divide robots into rule-based, cognitive, and self-learning. Autonomous virtual robots can also be called intelligent virtual agents [57]. Rule-based robots operate based on predefined rules and instructions, so they are not very flexible. Cognitive robots use artificial intelligence and machine learning to function [58]. Self-learning robots also use the AI area of reinforcement learning. A very basic example of the function of such robots are voicebots or chatbots that interact with the user; these can be found, for example, in retailing [59]. Other features that these robots have include RPA (robotic process automation), where robots are designed to perform repetitive tasks [60]. Another function that these robots are suited for is monitoring and responding dynamically to the environment. For example, these can be sales agents who make purchases. A smaller group are virtual assistants and web scrapers, for example, who extract information from web pages. In this article [61], for example, they describe how such a method is used to collect information about natural disasters. The areas where robots are found can be seen as web environments, robots integrated into various applications, cloud environments, or perhaps robots integrated directly into the operating system of a particular device.

#### 3.1.4. Robot Peripherals

In order to perform the tasks assigned to the industrial robot or even mobile robot, it is always necessary to use so-called peripherals. This is because peripherals include, for example, a gripper, without which the robot could not manipulate objects. A typical robot workcell includes one or more robots along with their controllers and various peripherals, such as grippers, tools, safety devices, sensors, and material transfer components, for handling and positioning parts [62]. Generally, the cost of a complete robot workcell is four to five times the price of the robots themselves. However, ongoing efforts aim to significantly lower these costs by leveraging enhanced robot functionality and advancements in artificial intelligence [63].

### 3.2. Communication Technologies

Communication technologies are a large part of the Internet of Things in general. They are used to transfer data between devices and to a central platform. Primarily, the user decides on specific technologies based on the application on which they are to be deployed. The decision-making process usually starts with the decision whether the transmission should be wired or wireless [64]. The basic differences are sorted in Table 1. Wired data transmission is usually carried out using cables that connect the different elements of the network. These cables provide a stable and reliable connection between devices, which is important for critical applications such as robots that require low latency and high data rates. Wired data transmission is also used in situations where higher data security and protection against interference is required. Typically, this type of data transfer is encountered in industrial applications. Here, we find that Ethernet or CAN, Modbus, Profibus, and HART are the most used [65]. In the article [66], we can get an idea as to whether these networks will be replaced by wireless ones. Wireless data transmission uses radio waves to transfer data between devices without the need for cables. This type of data transmission provides greater flexibility and convenience because there is no need to install cables, but it can also be prone to interference and signal disruption. Wireless data transmission is typically used in less critical applications, such as sensors, where low latency and high data rates are not required. The most significant ones will probably be the 5G network for some time to come. This [67] survey provides a comprehensive analysis of this network. In robotics, a combination of both types of data transmission is often used. For example, wired data transmission is often used for critical tasks such as robot control, while wireless data transmission is used for monitoring and collecting data from various sensors. However, modern high-speed networks like 5G [68] are slowly reducing the gaps between wire and wireless. Therefore, the need for wire-based data transmission is slowly diminishing, but it probably will not replace wired communication yet.

The complexity of data transfer in the robotic world using wireless technologies is best understood using layered models such as the Open Systems Interconnection (so-called as OSI) schema. As the name implies, the entire data transfer process is divided into several layers, each with a different task. These layered models are clearly described in this article [69], where their derivatives in the form of multi-layered models are also described.

#### 3.2.1. Communication Protocols

**Figure 4:**
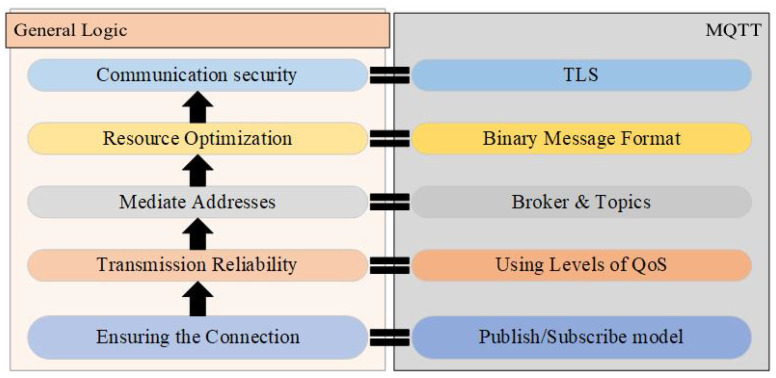
The principle of communication protocols with an example of the MQTT protocol.

The communication architecture serves as the backbone that allows devices to communicate with each other and exchange data [70]. The IoRT communication architecture works via the interaction of a multitude of components that together form the IoRT network [71]. Beyond the multitude of sensors, from mechatronic systems to cloud platforms, it is possible to find areas in the network that have seemingly marginal contributions but are now irreplaceable in the overall concept. We describe these areas as layers. Together, these layers form layered models that show us the complexity of the network in which data move [72]. The most basic layered model, which is also the simplest description of an IoRT network, is the three-layer model [73]. This model describes the lowest layer, called the physical layer, where data collection takes place in the IoRT concept through the network layer, where data transmission is realized, to the application layer, where data are processed. However, the simplicity of this model is limiting in more advanced areas; therefore, there are multi-layered models. The three-layer model is merely a description of the transfer of data from robotic systems to the cloud platform. For data transmission, we use some of the communication protocols as standard. We describe the principle of such protocols in Figure 4, which explains it using the MQTT protocol described below. In this subsection, we present some of the communication protocols used in IoT and robotics. For each, we present its deployment options and suitability. The first communication protocol is the MQTT (message queuing telemetry transport)protocol. It is a lightweight protocol that uses the publish-subscribe model. This model is suitable for bandwidth-constrained networks, as in this article [74]. It is also known for its low energy consumption. Apart from internet applications, the HTTP (HyperText Transfer Protocol) protocol is also used for data transfer between IoT devices [75], especially for applications where a large volume of data needs to be transferred. For example, it can be used to control a robot through a web interface. Similarity to this protocol can be found in the COAP protocol (Constrained Application Protocol) [76], which uses the UDP (User Datagram Protocol) for communication and, thanks to this, is not so demanding of computing power. It is used to transfer smaller amounts of data. Another protocol that can be found in most smart devices in the home—and not only there [77]—is Zigbee. This protocol is suitable for transferring data over short distances. Conversely, the LoRaWan (Long-Range Wide-Area Network) protocol is suitable for long-distance transmission. Due to its low power consumption, this protocol is suitable, for example, for monitoring premises and surroundings, typically in agriculture. LoRa (long-range) [78], powered by LoRaWan, is currently quite widely discussed. Another protocol that can be found in home systems is Z-WAVE [79]. It has a short range and low power consumption. Summaries, comparisons, and other protocols used can be found in this article [80] or in this article [81].

#### 3.2.2. Middleware Tools

Middleware in IoRT [82], as in IoT, is used to connect different devices and applications in the IoRT ecosystem [83]. Sometimes also referred to as a hidden translation layer, this layer enables efficient communication and data exchange between sensors, devices, applications, and the cloud. It can also be seen as an interface between IoT elements and the cloud. In addition to unifying data from different sources, it can also serve as a tool to bring existing systems into the network that were not originally designed for this purpose. Thus, in general, middleware serves to standardize communication and data formats in an IoRT network. This allows for greater interoperability and easier integration of different components in the IoT [84]. The interoperability problem is quite fundamental and an interesting solution can be found, for example, in this article by [85], where they propose their own middleware. Middleware software was originally created for on-premises devices, but today, thanks to the cloud trend, even middleware is more of a cloud solution. At the same time, there is also a large number of types of middleware. Among the most widely used is message-oriented middleware, which allows messages to be received and sent between distributed applications. There is also robotic middleware that is sized for the complexity of robotic systems, such as the robot operating system (ROS) [86], which provides a standardized framework for integrating various robotic components, managing data flow, and enabling communication between robots and other devices in a networked environment. The ROS offers a wide range of tools and libraries, including communication protocols, that support the development and integration of robotic systems, making it a crucial middleware for IoRT applications. The lightweight version of the ROS is the micro-ROS [87], which is designed for resource-constrained devices, such as embedded systems, which enables the use of ROS in low-power, memory-limited environments. The micro-ROS extends ROS capabilities to edge devices in IoRT networks, making it suitable for applications requiring smaller footprints while maintaining compatibility with the broader ROS ecosystem. Other examples include object middleware, which provides communication between objects, as well as database middleware, application server middleware, web middleware, and so on. Much of the middleware used in robotics offers interoperability, so it combines these capabilities into one software. Middleware often offers the possibility to build more complex applications on their basis, so they may include software development kits. The middleware applications are described in detail in Table 2.

### 3.3. On-Premises and On-Demand Solutions

There are a lot of things to consider when deciding between a cloud-based or on-premises solution. While the trend is definitely to move to the cloud, many companies are still staying true to on-premises solutions. If we look at both, we can easily see the main difference—location. While the cloud is stored and managed on the provider’s server and accessed through a web browser or other interface, an on-premises solution is run on local hardware infrastructure.

A company chooses on-premises software if it requires greater data security. This requires the purchase of a license for such software and the associated maintenance costs. It is also necessary to run in-house hardware that is sufficient for this solution. On the other hand, the user is not tied to an internet connection and the data are always available. Using in-house hardware can also provide a company with more flexibility and security for its data. In a cloud computing environment, the solution is not hosted in-house; instead, a third-party provider hosts everything for you. The solution is more flexible in terms of finances because the user sets the parameters according to the usage. The advantage is also global, i.e., accessing these resources from anywhere with only an internet connection. On the other hand, the internet connection can also be limiting, as the quality of the connection plays a big role in usage here. Data security is also lower; therefore, when operating with sensitive data, it is better to move the solution to be on-premises. Hybrid solutions that combine the good features of both work reliably.

### 3.4. “As-a-Service” Models

**Figure 5:**
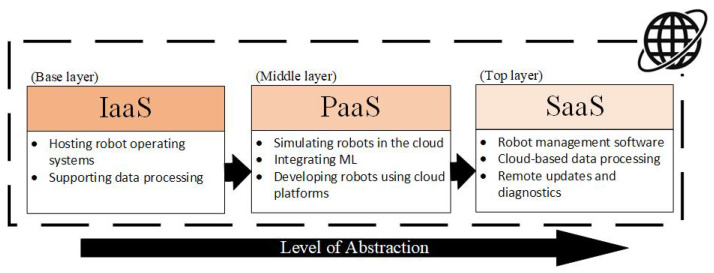
As-a-Service layered hierarchy.

“As-a-Service” (“aaS” for short) models are a way of delivering services over an internet connection. They allow the user to use applications and technologies without having to use their own hardware and equipment. The system works on the basis of cloud-based solutions and the user pays for their use. This way of working is nowadays very widespread due to the flexibility that these services bring. There are many variations of aaS, called XaaS (Anything as a Service), and they often differ only in small nuances. However, they are basically based on the three most commonly used ones. The hierarchy of the basic AaS models is described in Figure 5. Individual models carry the functions of their child models, but the child functions do not need to be operated by the user.

#### 3.4.1. Software as a Service

Software as a Service (“SaaS” for short) [88] is a model in which software is provided to the user via the internet. The software usually runs in a cloud environment that can be accessed remotely. This again carries potential security risks and risks of unavailability of the service in case of problems with the internet connection. Examples of such services in industry and robotics today are cloud-based design support software such as Fusion 360 from versions 2013 onwards.

#### 3.4.2. Platform as a Service

These models serve developers to a greater extent. They offer a complete cloud platform for developing, running, and managing applications. The big advantage of this model is scalability. Thus, the provider can easily and quickly scale the infrastructure to meet the demands of the application. This means, among other things, that the application can adapt to fluctuations in interest. As a result, it is then able to handle peaks in traffic. Among the most popular models is Heroku, which supports a wide variety of programming languages and provides a large number of tools and services for application development. In the context of IoRT, these models are the most significant. They make it possible to efficiently manage robotic systems and build robotic applications tailored to the requirements for monitoring systems and processes. At the same time, they can also replace the computational power of the robot, using applications that react to changes in the robot’s functioning or in its environment.

#### 3.4.3. Infrastructure as a Service

Infrastructure as a Service provides the building blocks for cloud infrastructure. It also provides computing resources such as computing power, virtual machines, and networking. Similar to the previous ones, the need for custom hardware is eliminated along with the need for management of these services. Examples of this model include Microsoft Azure or Google Compute Engine.

#### 3.4.4. AaS in IoRT

“As-a-Service” models, including Infrastructure as a Service (IaaS), Platform as a Service (PaaS), and Software as a Service (SaaS), provide a cloud-based framework that simplifies the development, deployment, and management of robotics and IoT applications. These models enable robotics and IoT systems to leverage scalable computing power, specialized development tools, and comprehensive software services without needing to manage underlying hardware or software complexities. In robotics, IaaS offers the computational resources required for tasks like simulation and data processing, while PaaS provides a development environment to build and test robotic applications integrated with IoT devices. SaaS facilitates real-time monitoring, fleet management, and analytics by offering end-user applications that communicate with both robots and IoT devices. In addition to the basic “As-a-Service” model described above, we can also find a number of other models. These include Function as a Service (FaaS), Data as a Service (DaaS), and Artificial Intelligence as a Service (AIaaS). FaaS enables the execution of small pieces of code in response to events without server management, with billing based only on runtime, making it a scalable and cost-effective solution. DaaS provides data on demand via the internet, with centralized management and scalability, and is commonly used for market analysis, IoT, or customer insights. AIaaS offers access to AI tools like machine learning and natural language processing without the need for in-house development, allowing businesses to easily integrate AI into their operations. By combining “As-a-Service” models with IoT, robotics systems can achieve seamless connectivity, remote control, and data-driven insights, leading to more intelligent, efficient, and scalable robotic solutions that operate autonomously and interact dynamically with their environments [89].

### 3.5. Software Tools

This section focuses on key tools used in the fields of IoT and IoRT, with particular emphasis on data analysis and machine learning tools that form the foundation of modern automation and robotics. Data collection and analysis are critical for the proper functioning of these systems, serving both immediate responses to changes in monitored parameters and long-term monitoring for predictive purposes, which also supports the development of machine learning models. The section delves into data analysis tools and advanced machine learning models, which are increasingly applied to enhance autonomous robot control, object detection, and diagnostics and maintenance. Given the continuous advancement of technology, understanding the latest tools and their applications is crucial for efficient processing and analysis of large data volumes in real time, as well as for predictive maintenance of robotic systems.

#### 3.5.1. Data Analysis Tools

One of the pillars of IoT—and therefore IoRT—is data gathering. Data collection is used both for immediate system responses and long-term monitoring. In immediate reactions, the system responds to a sudden change in the monitored parameter. For example, it can be a change in temperature, zone violations, loss of signal for some subsystems, etc. The response can be in the form of an alert to the user or an autonomous response by the robot. In this case [90], it is used by the fire robot radar to detect obstacles and to navigate through locations with poor visibility. Long-term monitoring data are then used to predict situations and are essential for machine learning applications. These tools leverage advanced algorithms, including machine learning and artificial intelligence, to extract meaningful patterns and insights from large datasets. For IoT systems, analytics tools often monitor real-time data streams, enabling anomaly detection, predictive maintenance, and optimization of device performance. In robotic systems, such tools analyze sensor data, operational metrics, and user interactions to improve decision-making, enhance efficiency, and ensure reliable operation. There are a number of tools to analyze data in detail, like MATLAB [91], and some that have already been implemented in IoT platforms. In addition, there are specific services in relation to “as-a-Service” models, such as DaaS or Big Data as a Service (BDaaS), for which the operator offers management or analysis of large data in data warehouses. In this paper, for example, Ref. [92] proposes a DaaS positioned between the Internet edge and the cloud, taking advantage of the benefits of both.

#### 3.5.2. Machine Learning Tools

The field of machine learning (referred to as ML) is nowadays widely used in the field of automation, and therefore also in the field of robotics [93]. A large number of applications are also devoted to the application of LLMs to [94] robotic systems. An LLM runs in the background of the ChatGPT model. Machine learning models are also used for object recognition, where the robot learns to recognize different objects based on their properties, which can be used, for example, for pick and place operations [95]. However, it is also used for autonomous robot control, an area used for collaborative robots. Localization is an important issue for mobile robots in terms of navigation; this paper [96] provides an overview of the available technologies for localization and offers insights into the future involvement of ML tools for solutions. Outside of these areas, machine learning is advantageous in terms of diagnostics and maintenance. Here, these tools can detect faults or estimate the lifetime of components. Among the machine learning tools used are TensorFlow [97,98], which is an open-source library that allows users to create and train neural networks and other machine learning models. TensorFlow was designed by Google and is a versatile tool for basic and advanced work. It is possible to use Keras [99]. It is an open-source Python library for developing and training neural networks. It has an interface to TensorFlow or PyTorch, which perform the training. Another open-source framework is PyTorch [100], developed by Meta AI. Its main advantage is its intuitive interface for creating machine learning models. A similarly user-friendly environment is offered by the Scikit-learn [101] library, which provides tools for preprocessed data and model evaluation. Scikit-learn also includes a number of tools for classification, regression, and dimensionality reduction. The same tools are offered by the open-source project Weka [102], which also has a user interface and thus facilitates working with models. At the same time, it also has data visualization capabilities. A detailed comparison of these tools can be found in Table 3.

These ML tools are written using programming languages that have a significant impact on the functionality of the tools; their comparison can be found in Table 4. These are mostly performance and speed parameters. The suitability of the programming language used is then largely determined by the nature of the task. The programming languages widely used to work with machine learning tools are Python [103] and the R [104] programming language. In general, the most widely used programming language for machine learning is Python, which is easy to apply and offers a large number of libraries and tools thanks to the broad support of the community. While the R programming language is designed for statistics and data analysis. Among the interesting programming languages that are used not only in ML but also for data mining is the relatively new Julia [105], which is, for example, significantly faster than Python.

## 4. Key Applications and State of the Art

**Figure 6:**
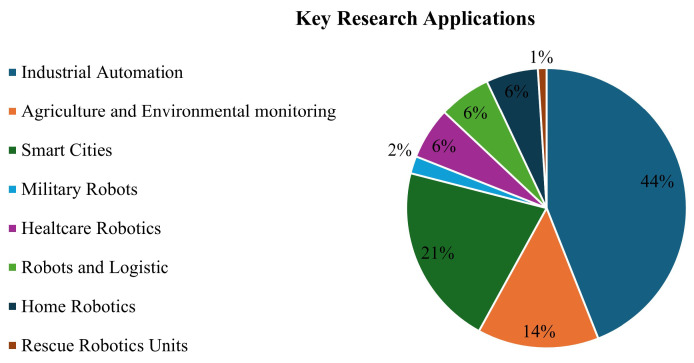
Research interest in Key Applications.

This section focuses on key applications where IoT and robotics intersect, describing their use across various industries such as industrial automation, healthcare robotics, agriculture, and logistics. For each sector, we will present specific examples of how IoT and robotics enhance efficiency, precision, and automation capabilities. Based on publication statistics from Scopus, which we describe in Section 2, and with the addition of keywords for each application area, we constructed a pie chart describing the research interest for each sector, as shown in Figure 6. Additionally, the section provides an overview of the current state of the art in these applications, highlighting recent scientific advancements and technological innovations, while addressing the challenges and opportunities that lie ahead for the development of these solutions.

### 4.1. Industrial Automation

**Figure 7:**
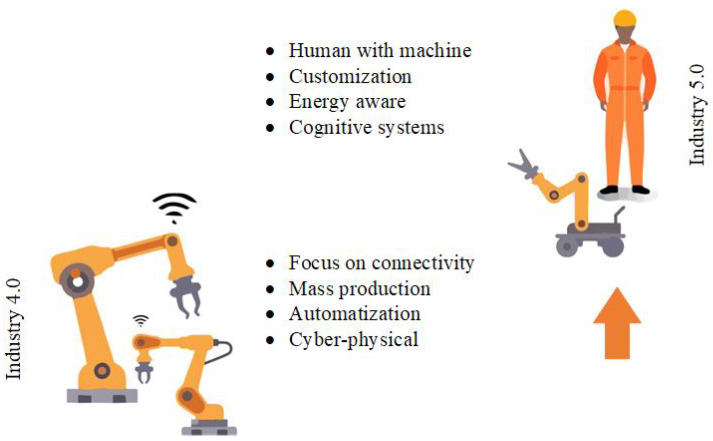
Main differences between Industry 4.0 and 5.0 approaches.

In industry, these systems are found both as peripettes for automatic lines and integrated directly into these systems. IoT systems integrated directly into production lines are mainly used for monitoring the status of machines, robots, and production systems [106]. For example, the [107] overview article offers an insight into the issues and approach to monitoring tool condition. It focuses, among others, on real-time monitoring and the use of IIoT technologies together with ML tools. These approaches enable us to plan maintenance and optimize production processes in a predictive manner. Automation of logistics and warehouses [108] is also used in the automated industry. Robotic systems using IoT are used for this purpose. These systems can be integrated into more complex systems and the entire logistics system can be much more efficient. The chapter [109] in this book discusses, among other things, applications in the Internet of Robotic Things in the industrial sector. The implementation of these systems in industry and manufacturing systems is ushering in an era of flexible and adaptive manufacturing [110], which is significantly supported by the emergence of wireless IoT technologies [111]. We are also gradually moving from Industry 4.0 to Industry 5.0 [112], which deals with issues such as carbon footprint, personalization and hyper-customization, human–robot cooperation, and predictive maintenance. The fundamental differences are described in Figure 7, with the main difference being the focus, where, unlike Industry 4.0, Industry 5.0 is human-centric. The number of these systems and their development is also enhanced by the support of deep learning tools [113].

### 4.2. Smart Cities

One of the main applications is autonomous transport [114], where robotic vehicles equipped with sensors and IoT systems enable safe and efficient movement around the city, including autonomous cars, buses, and drones for transporting goods or monitoring infrastructure [115]. One example is delivery services using robotic systems for last-mile delivery [116]. There are also applications such as smart lighting and energy systems, where smart systems and robots monitor energy consumption, which can be used for smart lighting on highways [117] or in public spaces. Similar applications can be found in all common experiences, such as the use of occupancy sensors that detect people in buildings [118] and are thus able to intelligently manage, for example, electricity consumption. In security, the combination is used for the monitoring and surveillance of public spaces, where autonomous robots and drones detect dangerous situations and support crisis intervention. Waste management is also benefiting from this technology [119], with autonomous robots monitoring the status of bins and optimizing their emptying, increasing the efficiency of waste management. Last but not least, smart buildings and infrastructure are controlled by IoT and robotics [120], enabling energy and water optimization, monitoring buildings, and performing predictive maintenance. Another interesting direction is the use of federated learning [121,122] in smart cities, where large amounts of data make it advantageous not to use centralized systems. These applications, and especially if they are supported by ML technologies [123], can make a significant contribution to creating the smart and sustainable cities of the future.

### 4.3. Agriculture and Environmental Monitoring

**Figure 8:**
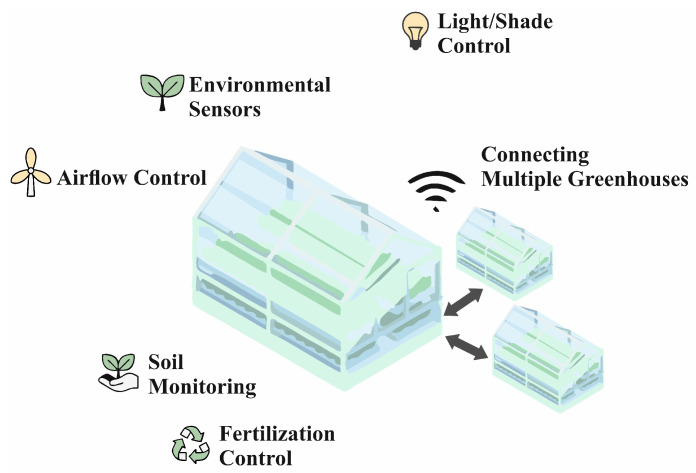
A smart greenhouse can significantly increase its productivity by integrating various sensor and robotic systems in combination with IoT.

In precision agriculture [124], IoT sensors and robots are used to monitor soil conditions, moisture, temperature, and other key parameters, enabling more accurate irrigation, fertilizer application, and crop protection, optimizing yield and reducing costs. This does not only apply to greenhouses, as shown in Figure 8. Given the nature of agriculture and the environment in general, we have to deal with data transmission when using IoT technologies [125,126]. What impact 5G networks will have on farming, for example, can be read in this article [127]. Autonomous farm machinery, such as tractors and harvesters equipped with IoT technologies, can operate more efficiently and with less human intervention, helping to automate the entire growing and harvesting process. In the field of environmental monitoring, IoT sensors and robotic systems enable the collection of real-time data on air, water [128], and soil quality, helping to identify environmental threats such as pollution or climate change early on. Similarly to the other areas studied, we find a large number of applications using ML [129], precisely because of the data it collects. Drone UAVs (Unmanned aerial vehicles) with integrated IoT systems are used to monitor forests [130], fields, and other natural areas, where they can monitor vegetation health, detect pests, or even carry out reforestation by planting seeds. Smart irrigation systems that use data from IoT sensors and automated robots optimize water consumption and help conserve natural resources. These technologies thus contribute significantly to a more sustainable and efficient use of natural resources, which is crucial for both agriculture and environmental protection.

### 4.4. Healthcare Robotics

Smart healthcare systems use IoT sensors to continuously monitor patients in real time, allowing doctors to monitor vital signs, which, as this study [131] describes in their proposed solution, would be advantageous for checking on elderly patients, for example, and even for times of limited patient visitation. Elderly care will be very important in the future as the population ages, with people now living to older ages; robots and IoT technologies will play a big role in this area. As healthcare itself is already benefiting from IoT technologies [132], when combined with robotics, it can bring about a fundamental transformation in this sector. Robotic surgery [133] is another important example where IoT-enabled robots enable complex surgeries to be performed with high precision and minimal risk to the patient, often remotely thanks to telemedicine technologies. In this [134] study describing the evolution of these systems over ten years, you can read, among other things, about the challenges facing the sector. We must also mention the field of micro- and nano robotics [135], which are rapidly developing and warrant close attention. A large number of endoscopic procedures today are also testing the use of soft robotics [136]. Of course, you can also find robots in the field of rehabilitation and assistive technologies [137]. Robotic exoskeletons and prostheses equipped with IoT sensors are being used to help patients with limited mobility better control their movements and speed up the rehabilitation process, such as in this application of an exoskeleton, described in this [138] study, for upper limb rehabilitation. Autonomous robots in hospitals and healthcare facilities can perform a variety of tasks, such as delivering medication, sterilizing rooms, or transporting patients, reducing the burden on healthcare staff and increasing operational efficiency. Smart pharmacies and dispensing systems, equipped with IoT sensors and robotic elements, can automatically prepare and administer medications with high accuracy, minimizing dispensing errors.

### 4.5. Robotic Rescue Units

A small group is the robotic rescue forces, which includes search and rescue (SAR) robots [139]. IoT systems allow these robots to collect and share real-time data with control centers and other rescue units. They can monitor the environment using sensors such as cameras, heat detectors, chemical sensors, or LIDAR to map the terrain. Linked systems also provide communication between multiple robots and synchronize them in the field. These systems allow operators to reach hard-to-reach or dangerous places. It is also necessary for robotic rescue units to address ethical issues [140]. We expect this area to grow in the future, given the tendency to replace humans in hazardous environments with robots.

### 4.6. Home Robotics

Smart home assistants, such as IoT-equipped robotic helpers, can automate various tasks such as cleaning, cooking, or home management [141]. For example, IoT sensor-equipped robotic vacuum cleaners and cleaning robots can map the home, plan optimal routes, and automatically keep floors clean. IoT-connected smart thermostats and lighting allow automatic temperature and lighting control based on the presence of people or time of day, resulting in energy savings and increased comfort. Security systems using a combination of IoT and robotics can monitor a home in real time using cameras, motion sensors, and autonomous guard robots to detect dangerous situations such as intrusion or fire. Health and care robots in smart homes can monitor the health of residents, especially the elderly, and provide basic care, including administering medication or monitoring vital signs, increasing their independence and safety [142]. Smart appliances, such as refrigerators, washing machines, or coffee machines, equipped with IoT sensors and robotic functions, can communicate with each other, optimize energy consumption and ensure the smooth running of the home.

### 4.7. Robotics in Logistics

**Figure 9:**
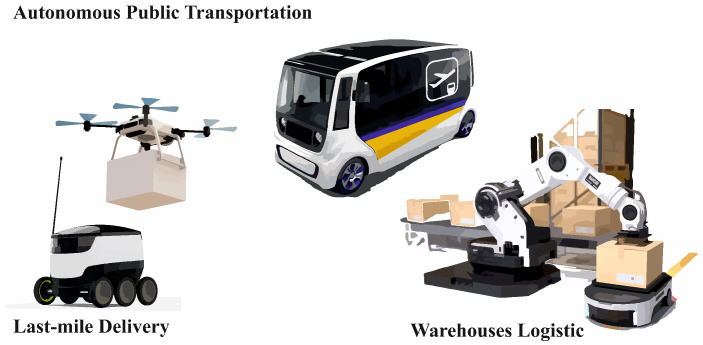
Division of robots in logistics according to our understanding.

Autonomous transport vehicles such as drones and self-driving vehicles equipped with IoT sensors enable efficient delivery of shipments, real-time monitoring of the traffic situation, and route optimization, which significantly reduces costs and increases delivery speed. Automated warehouses are another key application where robots control IoT systems to perform tasks such as sorting, retrieving, and transporting goods within the warehouse, increasing efficiency and minimizing errors [108]. Smart shelving and warehouse systems equipped with IoT sensors can monitor inventory levels in real time and automatically alert when replenishment is required, ensuring smooth operations and optimizing storage [143]. Robotic arms and other automated machines in distribution centres can pack and sort goods much faster than humans, increasing productivity and reducing labor costs. In the area of predictive maintenance, IoT sensors integrated into logistics robots can monitor their performance and alert the need for service before a breakdown occurs, minimizing downtime. Furthermore, smart transportation systems are able to optimize the overall transportation of goods by monitoring traffic congestion, weather, and other factors, allowing vehicles to be dynamically rerouted for the most efficient delivery. The group of robotic systems is divided into three smaller subgroups, as shown in Figure 9.

### 4.8. Robotics in the Military

Research on robotics and IoT for military purposes is often classified and not accessible to the public due to security reasons, but some applications of these technologies are known and show how they contribute significantly to increased efficiency and safety in military operations. Autonomous combat vehicles and drones, equipped with IoT sensors, allow reconnaissance and combat operations to be conducted without direct human involvement, reducing risk to soldiers. These vehicles can collect real-time battlefield data, communicate with other units, and autonomously react to changes in the situation, increasing the accuracy and speed of decision-making. Another important application is battlefield logistics, where autonomous robots and drones deliver supplies, ammunition, or medical material to combat operations zones, even in dangerous and hard-to-reach areas. These systems are controlled by IoT and ensure fast and safe delivery of the necessary equipment. Robotic exoskeletons equipped with IoT technologies are another example where military personnel can be equipped with assistive technologies that increase their physical capacity and allow them to carry heavier loads or move better in difficult terrain. Autonomous reconnaissance and surveillance drones can provide continuous battlefield monitoring, helping to detect enemy troops early, analyze terrain, and provide valuable intelligence. Cybersecurity systems integrated with IoT and robotic elements can monitor and protect military communications and information networks from cyber attacks, ensuring the security of digital infrastructure.

### 4.9. State-of-the-Art and Current Research Trends

In Section 3, we discussed the foundational logic guiding our approach to the problem. Based on this logic, we recognize that the baseline layer of our model involves data collection. Current advancements in research focus on deploying sophisticated sensing and perception technologies, particularly in robotics. This encompasses both industrial and mobile robots. For industrial robots, the emphasis is on enhancing human–robot collaboration and ensuring operational safety. For mobile robots, similar goals are pursued, with additional focus on improving spatial orientation, environmental adaptability, and autonomous learning. Advanced vision systems play a critical role here, enabling robots to gather extensive environmental data for more efficient operation like navigating through space [144]. However, processing such large volumes of data requires significant computational power, introducing a critical need for distributed computing solutions like edge and cloud computing.

Edge computing allows localized, real-time processing near the robotic device, reducing latency, which is critical for immediate responses. However, its limited computational capacity makes it less suitable for intensive tasks such as long-term monitoring or deep data analysis. Conversely, cloud computing offers substantial resources for complex computations and flexible scalability, making it ideal for tasks requiring heavy processing and storage. The integration of these computing paradigms supports collaborative robotics, particularly in multi-robot systems like drone swarms. Such scenarios involve multi-robot coordination, human–robot collaboration, and swarm robotics [145], which coordinates numerous simple robotic systems to achieve complex tasks.

Nonetheless, transferring data outside robotic systems raises significant concerns about data security and privacy. These challenges are particularly pertinent in IoT and collaborative robotic systems, where secure and reliable data exchange is crucial. Ensuring robust security measures in data transmission remains a priority, as highlighted in contemporary research addressing these challenges.

## 5. Current Challenges and Open Issues

We will divide the current challenges and problems in this area into several groups. The first and fundamental one is technical. Technical issues fundamentally affect the computational power for applications and are also limiting in terms of integrating IoT devices on robotic systems. The challenge in this group is then interoperability and integration of multi-robot systems. Another important group is data security and privacy concerns. With the increasing number of robotic devices operating with IoT devices, it is necessary to secure sensitive data. Security is directly related to the networking group. Beyond security, there is also the need to address suitability for individual robotic applications. Especially for mobile robotic systems using IoT technologies, the issue of energy management needs to be addressed. The integration of AI elements, the rise of which is evident, also poses challenges. Last but not least, we as a society need to address political and ethical issues.

### 5.1. Technical Challenges and Integration

**Figure 10:**
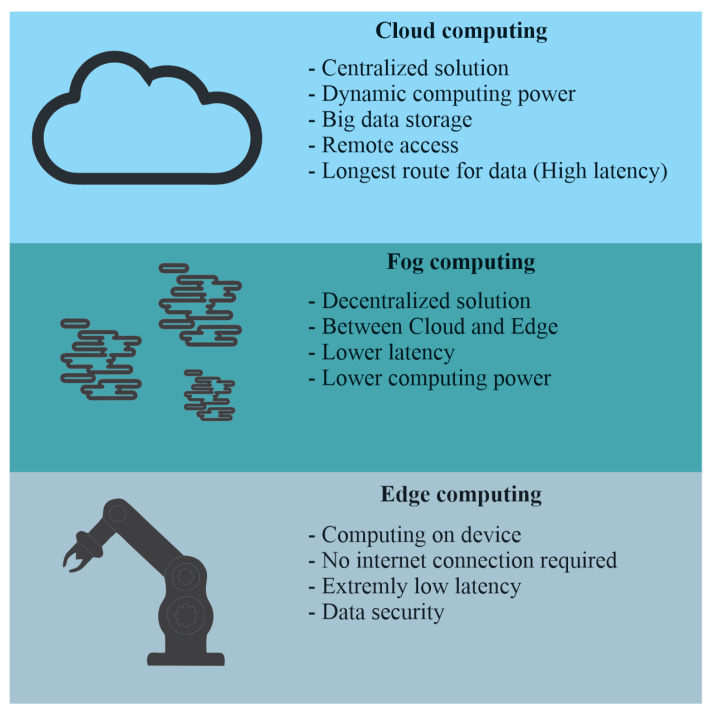
The roles of cloud, fog, and edge computing in the Internet of Robotic Things.

Combining robotic systems with IoT technologies or integrating IoT into robotics brings a number of complications and challenges. By nature, robotic systems are diverse and complicated systems, and the complexity of integrating IoT elements will depend on this. The article [146], for instance, describes the challenges in integrating the additive manufacturing industry with IoT and I4.0 technologies. Then, major challenges come in the area of interoperability, which we can simply describe as how to ensure a stable collection and data flow from different devices with different operating systems and sensor technologies combined into one centralized system, such as the cloud. The problem is solved, at least partially, by middleware, about which we wrote in Section 3.2.2. An interesting section of the fusion of robotics and IoT is multi-robotic systems. This section presents many challenges such as coordinating systems, ensuring stable communication, and/or scalability, i.e., ensuring that the system can handle an increasing number of robotic systems without losing efficiency and responsiveness. A summary of these issues is provided by the paper [147]. With increasing demands on robotic units—and even multi-robot systems—come challenges in computing power and big data processing. From Section 3.3, we know that we can have computing power located at the location where we need it, or at a more remote location, where access is mediated by the internet, which is called an on-demand solution. This part can then also be divided, including in terms of where the computation occurs, into edge computing, fog computing, and cloud computing. For applications requiring extensive computation and minimal latency (e.g., image processing), it is necessary to implement edge or fog computing to enable data processing as close to the source as possible. In this article [148], for example, you will learn which trends have been observed recently in edge computing. The next article [149] describes how fog-based architectures can help manage the increased data volumes that are generated by IoT technologies in industrial environments. The case study of this article [150] presents the use of cloud-based computing. A clearer description of the described techniques is shown in Figure 10. We also have to mention that, at the end of all these issues and challenges, there is one fundamental issue—whether it is cost effective.

This section concludes that integrating IoT technologies into robotic systems presents significant technical challenges, including ensuring interoperability, effective data processing, and system scalability. Middleware solutions and advanced computing paradigms like edge and cloud computing are key to addressing these issues. Ultimately, the success of such integration depends on achieving a balance between technical efficiency and cost-effectiveness.

### 5.2. Security and Privacy Concerns

Security and privacy are key concerns in robotics, especially in conjunction with the IoT. Robotic systems connected via the IoT collect, analyze, and share vast amounts of data, which may include sensitive information such as personal, location, or environmental data. In addition, they can also transfer important data related to corporate know-how, which is, among other reasons, why a large amount of the private sector is still based on on-premises solutions. IoT systems are often at risk of cyber-attacks such as unauthorized access, eavesdropping, or data manipulation, which can lead to security breaches not only for the robots but also for their users. For example, this article [151] describes some of the weaknesses of IoT applications, what attacks can target them, and how to defend against them. Another paper [152] talks about a mechanism for securing human-to-thing communication via HTTP and MQTT protocols in IoT. This paper [153] provides an overview of the challenges posed by the connectivity of IoT and robotics, while urging designers of intelligent robotic systems to consider the detection and prevention of attacks targeting their system. Blockchain technology [154] can also play a crucial role in enhancing the security and privacy of IoT-enabled robotic systems. By providing a decentralized and immutable ledger, blockchain ensures secure data sharing, making it harder for attackers to tamper with or manipulate critical data. It also enables transparent tracking of system activities, enhancing accountability. Prevention is also needed in terms of software and firmware updates for older devices. Communications between systems need to be encrypted in relation to data sensitivity, and the same applies to hardware, which needs to be adequately protected. Ensuring security includes data encryption, strong authentication mechanisms, and regular software updates to prevent misuse. At the same time, there are privacy concerns where people can be tracked or monitored without their knowledge, raising questions of ethics and legal liability. Balancing technological advances and privacy protection is therefore essential for the safe and responsible use of robotics and IoT. A summary is provided by the article [155], in which the authors call, among other things, for the implementation of robust cybersecurity measures and the promotion of cooperation between the parties involved, which can increase the resilience of robotic systems and reduce the impact of threats on society.

This section highlights the critical importance of security and privacy in IoT-enabled robotic systems. These systems handle vast amounts of sensitive data, making them vulnerable to cyberattacks and unauthorized access. Key challenges include securing data transmission, ensuring robust encryption, and protecting against software vulnerabilities. Additionally, ethical concerns arise regarding user privacy and the responsible handling of collected data. The section concludes by emphasizing the need for strong cybersecurity measures, regular updates, and collaboration among stakeholders to enhance system resilience and safeguard sensitive information.

### 5.3. Networking

**Figure 11:**
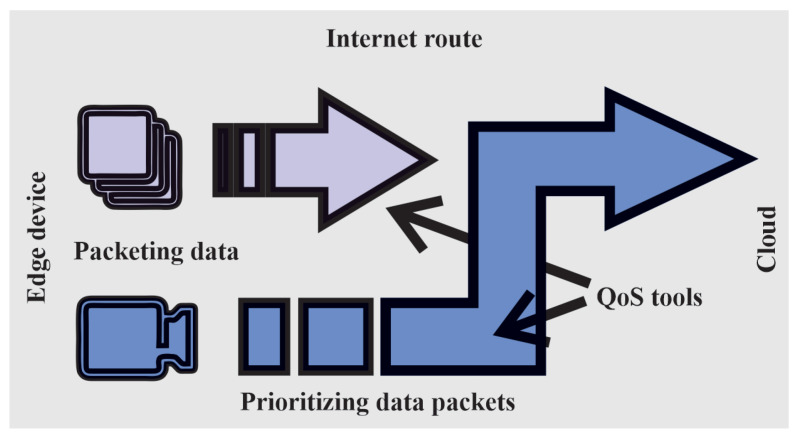
The role of QoS on the network is to prioritize more important data.

The previous section discusses the security risks, challenges, and areas that are central to the secure operation of IoRT systems. All are directly related to the operation of the network. In general, for different applications, we talk about the so-called QoS (quality of service). This indicates, in simple terms, what the network is good for, e.g., whether it is sufficient for image transmission and with what parameters. Other parameters depend on this value. The article [156] provides an insight into QoS optimization in smart healthcare. It offers a number of methods to achieve better results and to meet the needs of this environment. Next, the article [157] offers a method for guaranteeing QoS in real-time traffic. Another article [158] provides an overview of methods to optimize QoS. For multiple devices, we will also be interested in scalability, because increasing the number of devices puts a strain on the network, especially its bandwidth and latency. For this reason, it is advisable to have a proper connection management setup. In this paper, Ref. [159] describes the wireless communication of multi-robot systems (MRS), and also presents an application of cooperative transport using robots. Middleware and network operations in fog play a big role; here, we deal with the unification of information from different sources so that we centralize the unified information. To put this into context, when we talk about latency, we are talking about the delay in the network when transferring data. The delay can be unidirectional or bidirectional. We mainly deal with latency in applications where near-real-time data transfer is desired. For many applications where the data are not needed at this speed and at such high intervals, it is not so crucial. Another indicator is bandwidth, which tells us how much the network can carry, i.e., how much data can be transferred at a moment. In robotic environments, we also investigate the signal strength, by which we can get an idea of the reliability of the network. QoS also plays a key role in ensuring the prioritization of data packets between robots, sensors, and other devices in the network, enabling efficient and fast information exchange [160]. The article [161] provides insights into QoS optimization in multi-robot systems, offering methods to achieve better results and meet needs in this environment, which is particularly relevant when combined with ROS for managing inter-robot communication. In this way, QoS ensures that critical operations, such as robot control or event detection, are prioritized over less important traffic see Figure 11.

This section focuses on networking challenges in IoT-enabled robotic systems, emphasizing the need for reliable, scalable, and low-latency communication. Key aspects include optimizing quality of service (QoS) to meet the demands of real-time applications and managing bandwidth as the number of connected devices grows. The section also highlights the importance of robust connection management and middleware solutions to unify data from diverse sources. Ultimately, effective network optimization is critical to ensuring seamless operation and scalability of IoT-robotic systems.

### 5.4. Energy Consumption

Energy management is essential for the operation of all the technologies mentioned in this article. Key issues include optimizing energy efficiency, particularly for equipment performing computationally and mechanically demanding tasks, and ensuring minimal energy loss during idle periods. Limited battery capacity and the need for fast or wireless charging are further obstacles, along with the complexity of integrating renewable energy sources such as solar panels. This is particularly relevant for mobile robotic devices [162]. Centralizing data in the cloud partly addresses energy insecurity. Beyond that, securing what happens to the data in the event of a power outage is also a major issue. Thus, some backup systems or backup power sources needs to be addressed.

### 5.5. AI Integration

One of the main challenges is the complexity of combining AI and IoT in real time. This problem is partly solved by edge-AI integration, which allows data to be processed locally by the robots instead of being sent to the cloud. This allows for faster response times, reduced latency, and increased privacy, as data do not need to be transmitted over the internet. Edge-AI in robotics allows robots to perform tasks such as navigation, object recognition, and adapting to the environment in real time with greater efficiency. However, edge-AI must address limited computing power and memory on the devices themselves, which can limit the complexity of AI models that can be deployed. Another challenge is ensuring the reliability and accuracy of real-time decision-making, especially in dynamic and unknown environments. The article [163] provides an insight into the integration of AI into robotic process automation (RPA) [164], which has evolved into today’s form of intelligent process automation (IPA). RPA refers to software robots designed to automate repetitive, rule-based tasks by mimicking human actions within digital systems. These robots interact with user interfaces just like humans would, performing tasks such as data entry, file transfers, or processing invoices. On the other hand, IPA represents the next evolutionary step of RPA by incorporating advanced technologies such as AI, ML, and natural language processing (NLP). This combination enables IPA to handle more complex processes, work with unstructured data, and make intelligent, context-aware decisions. Coming back to automated greenhouses, Ref. [165] gives an insight into the current state of the art in relation to AI integration. AI enables robots to make autonomous decisions based on data collected from sensors and IoT networks, but this requires significant computing power and fast processing. Slow or inadequate responses can lead to operational errors, which is particularly critical in applications where safety is critical, such as industrial, healthcare, or rescue operations. Another challenge is the transparency and explainability of AI decision-making processes. AI-equipped robotic systems can make complex decisions based on neural networks and machine learning, making it difficult to understand how and why they reach certain conclusions. This raises liability issues, especially if an error or malfunction occurs that could endanger human lives or cause material damage. This is why AI-driven robots also raise ethical questions [166].

This section addresses the challenges of integrating AI into IoT-enabled robotic systems. It emphasizes the need for significant computational power and fast processing to enable real-time autonomous decision-making. Key issues include ensuring system transparency and explainability, particularly in safety-critical applications, and addressing potential ethical concerns related to AI in decision-making. The section concludes that successful integration of AI requires balancing technological advancement with safety, accountability, and ethical considerations.

### 5.6. Regulations, Policies and Ethnical Questions

One of the main challenges in regulation is the speed of technological development, which often outpaces the development of laws and standards. As a result, there is insufficient legal protection in areas such as liability for damage caused by autonomous robots or privacy protection in the use of IoT systems. For example, in accidents caused by autonomous vehicles or industrial robots, it is not always clear who is liable—the manufacturer, the operator, or the user. This question is still open and requires clearer definition at the policy and legal levels. Legal regulations for robotics and IoRT span international, regional, and national levels. Internationally, frameworks like the UN Charter and the Council of Europe’s Convention 108 provide a basis for protecting human rights, privacy, and safety. Regional regulations, such as GDPR (General Data Protection Regulation), NIS2 (Network and Information Security 2), and the proposed EU AI Act, define rules for data processing, cybersecurity, and accountability in autonomous systems. National legislation further addresses issues of liability, product safety, and data protection, filling gaps left by broader frameworks. Ethical issues play a crucial role, especially when it comes to privacy, surveillance, and data collection. Robotic systems and IoT often have access to sensitive information, whether it is data on health, user habits, or environmental monitoring. Here, an ethical conflict arises between technological possibilities and the protection of human rights, especially the right to privacy. Users should be informed about what information is collected, how it is used, and how it is protected from misuse. Transparency and user consent are key in this context. The [167] study provides an overview of the social ethics aspects of IoT. Principles such as those outlined by UNESCO and IEEE emphasize safety, accountability, and respect for human dignity and autonomy. This also applies to the application of AI in robotics; in this study [168], the authors describe the current state of the art on how to design trustworthy AI.

This section explores the regulatory, policy, and ethical challenges associated with IoT-enabled robotic systems. It highlights the need for updated laws to address liability, privacy, and safety concerns in rapidly evolving technologies. Ethical issues, such as transparency in data collection and user consent, are critical for maintaining public trust. The section concludes by emphasizing the importance of clear regulations, ethical frameworks, and collaboration among stakeholders to ensure the responsible and equitable development of robotics and IoT technologies.

## 6. Future Directions and Research Opportunities

The future of smart factories is closely tied to the advancement of IoT, robotics, and the integration of these technologies within dynamic production systems. Industry 5.0 [169], with its focus on human-centric manufacturing [170], offers exciting prospects for the evolution of human–robot collaboration, where machines and humans work together to achieve more adaptive, flexible, and personalized production. This convergence will be further enhanced by the widespread use of IoT technologies, enabling seamless communication between devices, systems, and workers in real time. IoT sensors and data analytics will provide valuable insights to optimize production processes, predict maintenance needs, and improve decision-making in smart manufacturing environments. As industries embrace these innovations, dynamic production systems will become more responsive to market demands, supply chain shifts, and other external factors. Additionally, the growing emphasis on energy efficiency and ecological sustainability in manufacturing will drive research into green technologies, including energy management, renewable sources, and resource-efficient robotics. The integration of robotics with IoT and eco-friendly practices presents numerous opportunities for the development of smarter, more sustainable factories [171]. Future research must explore how these technologies can be combined to create scalable, eco-conscious solutions that not only increase productivity but also minimize environmental impact.

Smart factories will not only increasingly rely on multi-robotic systems, where multiple autonomous or semi-autonomous robots collaborate to perform complex tasks in a highly synchronized manner. These systems, powered by advanced robotics, AI [172], and IoT technologies, require seamless interoperability to function effectively across diverse platforms and environments. Interoperability standards are essential for ensuring that different robots, regardless of manufacturer or design, can communicate, exchange data, and coordinate actions in real time. The development of common protocols and communication frameworks will be pivotal in enabling the efficient integration of robots into a unified system. Furthermore, multi-agent systems (MAS) will play a key role in orchestrating collaborative behaviors between robots, facilitating decentralized decision-making and problem-solving in dynamic production settings. By utilizing IoT, robots within a multi-robot system can share information, such as sensor data, operational status, and environmental conditions, leading to more informed and adaptive decision-making. This IoT-enabled collaboration allows for enhanced flexibility, scalability, and robustness in manufacturing operations, as robots can dynamically adjust their tasks and interactions based on real-time data. The ongoing research in these areas will focus on creating standardized interfaces and communication protocols, improving coordination algorithms, and ensuring that these multi-robotic systems can operate autonomously yet cooperatively in a wide range of industrial applications.

The integration of cloud, fog, edge, and quantum computing is essential for achieving the real-time processing, scalability, and flexibility required by modern robotics systems [173]. These computing paradigms enable the dynamic allocation of computing power, ensuring that resources are available where and when they are needed without overloading central servers. Cloud computing provides a centralized platform for large-scale data storage and analysis, while fog and edge computing push computation closer to the source of data generation, enabling faster decision-making and reducing latency in critical processes. Quantum computing, with its ability to process vast amounts of data simultaneously using quantum bits, could further enhance these systems by solving complex optimization and machine learning problems more efficiently, potentially revolutionizing the capabilities of robotics. With the advent of superfast networks such as 5G [174], 6G [175], and the implementation of IPv6, the capacity for high-speed, low-latency communication between devices, robots, and control systems is greatly enhanced, enabling seamless, real-time interaction across distributed manufacturing environments [176]. These ultra-fast networks will facilitate the rapid transfer of large data volumes and support the continuous exchange of information between machines and cloud infrastructures. Additionally, energy-independent devices are becoming increasingly vital, especially in environments where constant access to power is not guaranteed. Through the development of energy harvesting technologies and low-power designs, these devices can operate autonomously, drawing power from renewable sources or even the environment itself. Research in this area is expected to focus on optimizing energy management systems [177], improving network protocols, and creating more efficient computational models that can dynamically adapt to the needs of smart manufacturing systems.

Industry 5.0 marks a fundamental shift towards human-centered factories [178], where the emphasis is not just on automation and efficiency, but on enhancing the role of human workers through advanced technology. In this new era, factories will prioritize customization, enabling highly personalized production that meets the specific needs and preferences of consumers. Rather than being replaced by machines, human workers will collaborate more closely with robots and AI systems, leveraging their unique cognitive abilities to oversee complex decision-making processes and ensure quality control in dynamic production environments. This human-centric approach is supported by cognitive systems—AI-driven technologies that enable machines to learn, adapt, and make decisions in ways that are aligned with human values and priorities [179]. The interaction between human intelligence and artificial intelligence will be a defining characteristic of Industry 5.0 [180], enhancing creativity, problem-solving, and innovation within the manufacturing space.

While Industry 5.0 represents a new direction, it is important to note that Industry 4.0 will continue to evolve in parallel [181]. The foundation of Industry 5.0 is built upon the digital technologies introduced in Industry 4.0, including IoT, big data, AI, and robotics. As such, Industry 4.0 is not being superseded but rather extended and enhanced. The ongoing development of Industry 4.0 technologies, such as smart factories, digital twins, and autonomous systems, will complement the human-centric focus of Industry 5.0, creating a more integrated, flexible, and sustainable manufacturing ecosystem. Future research will likely explore how to bridge the capabilities of Industry 4.0 with the emerging needs of Industry 5.0, ensuring a smooth transition and the continued growth of both paradigms.

The implementation of artificial intelligence and machine learning is poised to drive significant advancements in smart manufacturing, offering the potential for more intelligent, efficient, and adaptive production systems. In the future, AI and ML algorithms will be deployed closer to the edge of networks, allowing for real-time data processing and decision-making directly at the source. This approach, often referred to as edge AI, will reduce latency, enhance privacy, and increase operational efficiency by minimizing the need to send large volumes of data to centralized cloud servers. AI-powered predictive maintenance, demand forecasting, and quality control systems will enable manufacturers to proactively address issues, optimize resources, and customize production lines at an unprecedented level.

However, as AI and ML technologies become more deeply integrated into industrial operations [93], ethical considerations must be addressed. Ethical AI will be crucial to ensure fairness, transparency, and accountability in decision-making processes. Issues such as algorithmic bias, data privacy, and the potential for unintended consequences must be carefully managed to ensure these technologies are used responsibly. Moreover, the implementation of robust policies and regulatory frameworks will be essential to govern the use of AI and ML [182]. Policymakers will need to create standards for data sharing, security, and the responsible deployment of AI systems to mitigate risks and ensure that the benefits of these technologies are broadly distributed. As AI and ML evolve, future research will likely focus on balancing innovation with ethical considerations, ensuring that their implementation leads to sustainable and inclusive advancements [183].

As smart manufacturing and robotic systems [184] become increasingly interconnected and reliant on advanced technologies like IoT, AI, and robotics, ensuring robust security will be critical to protect sensitive data, intellectual property, and the integrity of production processes [154]. Enhanced security will be a key focus of future research, with an emphasis on developing more resilient systems capable of proactively identifying and mitigating threats. Self-healing security is an emerging concept that involves the use of AI and machine learning to automatically detect security breaches or vulnerabilities and initiate corrective actions without human intervention. This autonomous response mechanism will help minimize downtime and prevent damage by allowing systems to recover quickly from attacks, making manufacturing processes more resilient to cyber threats.

Additionally, adaptive security will play a crucial role in safeguarding dynamic and evolving manufacturing environments. Unlike traditional security measures, which often rely on static rules, adaptive security systems will be capable of continuously learning from new data and threats, adjusting their defense mechanisms in real time. This approach ensures that security protocols can evolve alongside the rapidly changing landscape of industrial technologies. Future research in this area will likely focus on integrating self-healing and adaptive security mechanisms into IoT devices, robotics, and AI systems, creating intelligent, autonomous defense frameworks that can anticipate and neutralize threats before they cause harm. By advancing security technologies that are both proactive and responsive, researchers will help ensure that the benefits of smart manufacturing are realized safely and securely.

## 7. Conclusions

The integration of the Internet of Things with mobile and industrial robotics, forming the Internet of Robotic Things, represents a significant advancement in the field of industrial automation and beyond. This survey has highlighted the key technologies, applications, and challenges associated with IoRT. By leveraging IoT technologies, robotic systems can achieve higher levels of efficiency, autonomy, and intelligence, which are crucial for modern industrial applications.

The applications of IoRT span various sectors, including healthcare, agriculture, and manufacturing, demonstrating its versatility and potential to revolutionize these industries. However, the implementation of IoRT also presents several challenges, such as data security, energy efficiency, and ethical considerations, which must be addressed to fully realize its benefits.

Future research should focus on overcoming these challenges and exploring new applications of IoRT. By continuing to innovate and improve IoRT technologies, we can unlock new possibilities for automation, enhance productivity, and create smarter, more responsive systems that can adapt to the ever-changing demands of the modern world.

In conclusion, IoRT is poised to play a pivotal role in the future of robotics and automation, offering significant advantages and opportunities for various industries. As we continue to develop and refine these technologies, the potential for IoRT to transform our world becomes increasingly apparent.

## Figures and Tables

**Table 1:** Comparison of different data transmissions.

Criteria	Wire	Wireless
**Speed**	100–1000 Mbps	50–600 Mbps
**Stability (Variation)**	5%	20%
**Latency**	1–5 ms	10–30 ms
**Security**	Less secured	Secured
**Coverage**	100 m	70 m
**Interference**	Minimum	Inclined
**Cost**	Higher	Less
**Mobility**	Limited	High mobility

**Table 2:** Comparison of middleware used in IoRT.

Criteria		AWS IoT Core	Microsoft Azure	IBM Watson	Kepware	Oracle IoT Cloud	ThingsBoard	Mainflux
**Supports Multiple Protocols**	Yes	Yes	Yes	Yes	Yes	Yes	Yes	Yes
**Scalability**	Yes	Yes	Yes	Yes	Yes	Yes	Lim	Lim
**Edge Computing Support**	Yes	Yes	Yes	Lim	No	Lim	No	No
**Advanced Security Features**	Yes	Yes	Yes	Yes	Yes	Yes	Lim	Lim
**Compatibility with Cloud**	Yes	Yes	Yes	Yes	Yes	Yes	Lim	Lim
**Developer Tools and SDKs**	Yes	Yes	Yes	Yes	Yes	Yes	Lim	Lim
**Real-time Monitoring**	Yes	Yes	Yes	Yes	Yes	Yes	Lim	Lim
**Open-Source**	No	No	No	No	No	No	Yes	Yes

**Table 3:** Comparison of PyTorch, TensorFlow, Keras, Scikit-learn, and Weka.

Criteria	PyTorch	TensorFlow	Keras	Scikit-Learn	Weka
**Supports deep learning models**	Yes	Yes	Yes	No	No
**Supports training of CNN**	Yes	Yes	Yes	No	No
**Supports RNN**	Yes	Yes	Yes	No	No
**Export models to ONNX format**	Yes	Yes	No	No	No
**Supports deployment on mobile devices**	Yes	Yes	Lim	No	No
**Supports integration with Apache Spark**	No	Yes	No	Yes	No
**Integration with hyperparameter tuning tools**	Yes	Yes	Yes	Yes	No
**Supports distributed training**	Yes	Yes	Lim	No	No
**Provides pre-trained models**	Yes	Yes	Yes	No	No
**Compatible with Hugging Face library**	Yes	Yes	Yes	No	No
**Handles image data**	Yes	Yes	Yes	Yes	No
**Handles text data (NLP)**	Yes	Yes	Yes	Yes	No
**Supports time-series data**	Yes	Yes	Yes	Yes	Yes
**Integrated with TensorBoard**	No	Yes	Yes	No	No
**Supports training on GPU**	Yes	Yes	Lim	No	No
**Supports training on TPU**	No	Yes	Lim	No	No
**Supports training on Kubernetes cluster**	Yes	Yes	No	No	No
**Open-source**	Yes	Yes	Yes	Yes	Yes
**Allows commercial use (license)**	Yes	Yes	Yes	Yes	Yes
**Supports privacy-preserving ML**	Yes	Yes	No	No	No
**Includes security and fairness tools**	Yes	Yes	No	No	No
**Has GUI for non-coding users**	No	No	No	No	Yes
**Suitable for beginners**	No	No	Yes	Yes	Yes

**Table 4:** Comparison of programming languages suitable for machine learning.

Criteria	Python	R	Java	C++	Julia
**Speed (1—Fastest, 5—Slowest)**	3	5	4	1	2
**Parallel processing and distribution**	Yes	Lim	Yes	Yes	Yes
**Availability of ML/DL libraries**	Yes	Yes	Lim	Lim	Yes
**Pre-trained model availability**	Yes	Lim	Lim	No	Lim
**Tools for experiment tracking and visualization**	Yes	Lim	Lim	Lim	Yes
**Data engineering tools**	Yes	Yes	No	No	Yes
**Deployment support**	Yes	No	Yes	Yes	Lim
**GPU/TPU support**	Yes	No	Lim	Yes	Yes
**Distributed computing support**	Yes	Lim	Yes	Yes	Yes
**Data science tools**	Yes	Yes	Lim	No	Yes
**Industrial adoption**	Yes	Lim	Yes	Yes	Lim
**Open-source**	Yes	Yes	Yes	Yes	Yes

## Data Availability

The data that support the findings of this article are available from the corresponding author upon reasonable request.
